# Association of sex hormones and sex hormone-binding globulin with liver fat in men and women: an observational and Mendelian randomization study

**DOI:** 10.3389/fendo.2023.1223162

**Published:** 2023-10-13

**Authors:** Xinting Cai, Barbara Thorand, Simon Hohenester, Cornelia Prehn, Alexander Cecil, Jerzy Adamski, Tanja Zeller, Andrea Dennis, Rajarshi Banerjee, Annette Peters, Hanieh Yaghootkar, Jana Nano

**Affiliations:** ^1^ Institute of Epidemiology, Helmholtz Zentrum München, German Research Center for Environmental Health, Neuherberg, Germany; ^2^ Institute for Medical Information Processing, Biometry, and Epidemiology – IBE, Faculty of Medicine, Ludwig-Maximilians University of Munich, Munich, Germany; ^3^ Pettenkofer School of Public Health, Ludwig-Maximilians University of Munich, Munich, Germany; ^4^ German Center for Diabetes Research (DZD), partner site Munich-Neuherberg, Neuherberg, Germany; ^5^ Department of Medicine II, University Hospital, Ludwig-Maximilians University of Munich, Munich, Germany; ^6^ Core Facility Metabolomics and Proteomics, Helmholtz Zentrum München, German Research Center for Environmental Health, Neuherberg, Germany; ^7^ Institute of Experimental Genetics, Helmholtz Zentrum München, German Research Center for Environmental Health, Neuherberg, Germany; ^8^ Department of Biochemistry, Yong Loo Lin School of Medicine, National University of Singapore, Queenstown, Singapore; ^9^ Institute of Biochemistry, Faculty of Medicine, University of Ljubljana, Ljubljana, Slovenia; ^10^ University Center of Cardiovascular Science, University Heart and Vascular Center Hamburg, Hamburg, Germany; ^11^ Clinic of Cardiology, University Heart and Vascular Center Hamburg, Hamburg, Germany; ^12^ German Center for Cardiovascular Research (DZHK), Partner Site Hamburg/Kiel/Lübeck, Hamburg, Germany; ^13^ Perspectum, Oxford, United Kingdom; ^14^ German Center for Cardiovascular Disease Research (DZHK), partner site Munich Heart Alliance, Munich, Germany; ^15^ College of Health and Science, University of Lincoln, Joseph Banks Laboratories, Green Lane, Lincoln, Lincolnshire, United Kingdom

**Keywords:** sex hormones, sex hormone-binding globulin, fatty liver index, liver fat, Mendelian randomization, European cohort

## Abstract

**Background:**

Sex hormones and sex hormone-binding globulin (SHBG) may play a role in fatty liver development. We sought to examine the association of various endogenous sex hormones, including testosterone (T), and SHBG with liver fat using complementary observational and Mendelian randomization (MR) analyses.

**Methods:**

The observational analysis included a total of 2,239 participants (mean age 60 years; 35% postmenopausal women) from the population-based KORA study (average follow-up time: 6.5 years). We conducted linear regression analysis to investigate the sex-specific associations of sex hormones and SHBG with liver fat, estimated by fatty liver index (FLI). For MR analyses, we selected genetic variants associated with sex hormones and SHBG and extracted their associations with magnetic resonance imaging measured liver fat from the largest up to date European genome-wide associations studies.

**Results:**

In the observational analysis, T, dihydrotestosterone (DHT), progesterone and 17α-hydroxyprogesterone (17-OHP) were inversely associated with FLI in men, with beta estimates ranging from -4.23 to -2.30 [p-value <0.001 to 0.003]. Whereas in women, a positive association of free T with FLI (β = 4.17, 95%CI: 1.35, 6.98) was observed. SHBG was inversely associated with FLI across sexes [men: -3.45 (-5.13, -1.78); women: -9.23 (-12.19, -6.28)]. No causal association was found between genetically determined sex hormones and liver fat, but higher genetically determined SHBG was associated with lower liver fat in women (β = -0.36, 95% CI: -0.61, -0.12).

**Conclusion:**

Our results provide suggestive evidence for a causal association between SHBG and liver fat in women, implicating the protective role of SHBG against liver fat accumulation.

## Introduction

Fatty liver, a condition characterized by excessive ectopic fat accumulation in the liver (≥5%), is affecting one fourth of the world population. It is increasingly contributing to the global healthcare burden with the late stage of liver disease, liver cirrhosis, being the 11^th^ most common cause of death (1).

Epidemiological evidence reported that fatty liver is more prevalent among men than women (2). Several mechanisms have been proposed to explain these differences focusing mainly on the role of sex hormones, namely androgens and estrogen, on glucose-, cholesterol- and lipid- metabolism in the liver (3). Endocrine diseases such as male hypogonadism, a condition defined by reduced sex hormone levels, or polycystic ovary syndrome (PCOS), a condition usually resulting in excessive androgen levels in women, have been consistently shown to be associated with higher fatty liver risk (3).

A recent meta-analysis of population-based studies found that higher serum testosterone (T), the major form of androgen, was associated with lower risk of fatty liver among men, but not in women (4). Other studies on precursors of T such as dehydroepiandrosterone (DHEA) and its sulfate form DHEA-sulfate (DHEAS), have consistently shown an involvement in metabolic disorders (5). For example, supplementation of DHEA improved insulin sensitivity and increased lean body mass in older adults (6, 7). However, whether DHEA or DHEAS modulate fatty liver risk remains controversial (4, 8). In peripheral tissues, such as skin, DHEA and T are converted into dihydrotestosterone (DHT), and the latter has been related to lower risk of diabetes among older men (9). Nevertheless, there is no population-based evidence directly linking DHT to fatty liver.

Postmenopausal women exhibited higher fatty liver risk compared to premenopausal women, highlighting the protective role of estrogens, such as estradiol (E2), in cardiometabolic health (10). Other important sex hormones, such as progesterone and its derivative, 17α-hydroxyprogesterone (17-OHP), have also been linked to metabolic derangements, such as insulin resistance, obesity and diabetes (11, 12), conditions closely related to fatty liver (1). Sex hormone-binding globulin (SHBG), on the other hand, a liver derived protein that transports sex hormones in the blood and affects their bioactivity (13), has been associated with lower odds of fatty liver in both men and women in a recent meta-analysis (4).

In this study, we firstly aimed to investigate the cross-sectional and longitudinal association of serum sex hormone levels (e.g. T, DHEA) and SHBG with the fatty liver index (FLI), a validated non-invasive and cost-efficient tool for the estimation of fatty liver in population-based studies (14, 15). Secondly, to investigate whether the observed associations are causal, we used genetic instruments to investigate the role of sex hormones and SHBG on liver fat by Mendelian randomization analysis using the largest up to date genome-wide association studies (GWAS) (16–19).

## Methods

### Population

The study was performed among participants of the prospective population-based Cooperative Health Research in the Region of Augsburg (KORA) study. A total of 4,261 adults, aged 25-74 years, were included at baseline between 1999 and 2001 (S4 visit) with the primary aim to assess health and disease in Southern Bavaria, Germany. Follow-up examinations were conducted after 7 years (F4 visit, 2006 -2008) and after 14 years (FF4 visit, 2013 - 2014) (20–22). All study participants have provided written informed consent. The study was approved by the Ethics Committees of the Bavarian Chamber of Physicians (Ethical Approval Number 06068) adhering to the declaration of Helsinki.

**Figure 1:**
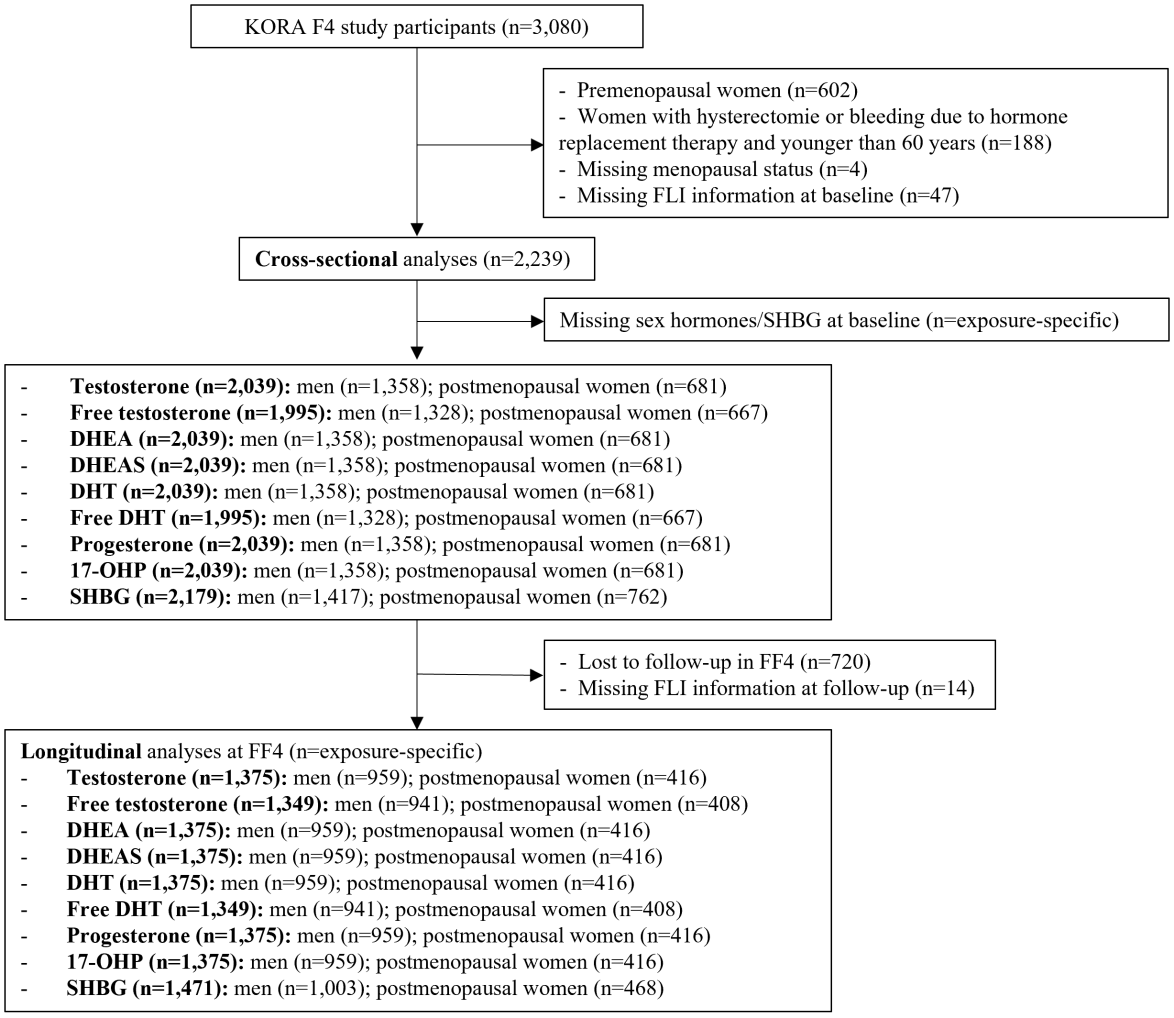
Flow chart of the KORA study population for the observational analysis. DHEA, dehydroepiandrosterone; DHEAS, dehydroepiandrosterone-sulfate; DHT, dihydrotestosterone; SHBG, sex horhome-binding globulin; 17-OHP, 17α-hydroxyprogesterone.

The present analysis includes data from the F4 visit as baseline and FF4 visit as follow-up (average follow-time: 6.5 years). Excluding premenopausal women (n = 602), women with hysterectomy or bleeding due to hormone replacement therapy and younger than 60 years (n = 188), women with missing menopausal status (n = 4), participants without valid FLI information at baseline (n = 47), a total of 2,239 participants (1,456 men and 783 postmenopausal women) were included in the cross-sectional analysis (**Figure 1**). Due to missing sex hormones information at baseline (n = 60 to 244), the final number of participants for the regression analyses differed by sex hormone (1,328 to 1,417 men; 667 to 762 postmenopausal women) at baseline. For the longitudinal analysis, we further excluded participants lost to follow-up (n = 720) and those without FLI information (n = 14) at the FF4 visit, leaving a sample size of 1,505 participants (941 to 1,003 men; 408 to 468 postmenopausal women).

Details of laboratory, clinical and anthropometric measurements as well as interviews are provided in the **Supplementary Materials**.

### Sex hormones and SHBG assessments

T, DHEA, DHEAS, DHT, progesterone, and 17-OHP were quantified in serum samples which were stored at -80°C until being assayed. The detailed assessment procedure has already been described in detail (23). Samples were prepared and sex hormones were quantified using the Absolute*IDQ*™ Stero17 Kit and electrospray ionization liquid chromatography-mass spectrometry (ESI-LC-MS/MS). The quantification method of the Absolute*IDQ*™ Stero17 Kit has been proved to follow the European Medicines Agency’s Guideline on bioanalytical method validation (July 21^st^ 2011) (24). Metabolite concentrations were calculated using internal standards and reported in nM or ng/ml. Missing values of sex hormones were imputed (11). Sex hormones were then normalized, and different batches were calibrated (11). SHBG was measured in serum using the chemiluminescent microparticle immunoassay ARCHITECT for the absolute quantification of SHBG (Abbott Diagnostics).

In order to be transported in blood, sex hormones are bounded to SHBG or weakly bounded to albumin. The free circulating sex hormones [e.g. free T (fT), free DHT (fDHT)] represent the bioactive hormones that target tissues. The sum of albumin-bound and free sex hormone is bioavailable sex hormone (e.g. bioavailable T). In the KORA study, fT and fDHT were calculated using mass action equations based on the concentrations of the total hormones and their binding constants to serum SHBG and albumin according to Rinaldi et al. (25).

### Calculation of FLI

FLI was calculated from BMI, waist circumference, triglycerides (TG) and gamma-glutamyl-transferase (GGT) with the algorithm developed by Bedogni et al. (14):

FLI = (e ^0.953*loge (TG) + 0.139*BMI + 0.718*loge (GGT) + 0.053*waist circumference - 15.745^)/(1 + e ^0.953*loge (TG) + 0.139*BMI + 0.718*loge (GGT) + 0.053*waist circumference - 15.745^) * 100, with TG measured in mmol/l, GGT in U/l, and waist circumference in cm, resulting in a score ranging from 0 to 100, with a FLI < 30 ruling out and a FLI ≥ 60 ruling in fatty liver.

### Genetic instrumental variables

We searched the GWAS Catalogue using the full name of the sex hormones and identified the largest GWAS in the European population including total T, bioavailable T (bioT), E2, SHBG (18, 26), DHEAS (17), progesterone and 17-OHP (19). For DHT, the only GWAS in the European population was conducted in a study population at particular high risk of prostate cancer (men only, n= 3225) (27). One GWAS identified a SNP (rs34670419) associated with DHEA in a European population (n=1023); however, the association (*p*=2e-9) did not reach a genome-wide cut-off of *p*<5e-11 after multiple-testing adjustment (28). Therefore, we did not include DHT and DHEA in the MR analysis. Summary statistics for total T in men and women, bioT in men and women, E2 in men, SHBG in men and women (Ruth et al., 2020 (18)), DHEAS in men and women combined (Zhai et al., 2011 (17)), and progesterone in men and women, 17-OHP in men and women (Pott et al., 2021) (19) were obtained from the respective publications. Of note, a genetic instrument for E2 in women was not included, as in the GWAS of Ruth et al. most of the women were postmenopausal and showed E2 levels below the limit of detection (78%), which substantially reduced the power of analysis for genetic instruments of E2 and biased the associations towards loci associated with age at menopause (18).

After we included the genome-wide significant SNPs (*p*<5e-8) for sex hormones and SHBG, we clumped the SNPs if they were in linkage disequilibrium (LD) (LD r^2^>0.001). The SNP-outcome associations were extracted from the largest GWAS available up to date for MRI measured hepatic proton-density fat fraction (PDFF) in the European population by Parisinos et al. using data from a subsample of UK Biobank (29) (**Supplementary Table 1**). We chose this study because MRI has been demonstrated as the most definitive non-invasive medical imaging to quantify liver fat content (30). Afterwards, we harmonized the SNP-exposure and SNP-outcome associations and excluded palindromic SNPs (**Supplementary Figure 1**). Three genetic instruments that could not be matched in the outcome dataset (rs543504257, rs2275560, rs78058190) were excluded from further MR analysis.

### Statistical analyses

Baseline characteristics of the participants were compared among the FLI categories stratified by sex. For continuous variables, the arithmetic mean and standard deviation (SD) are shown if normally distributed or the median and interquartile (IQR) if non-normally distributed. For categorical variables, counts and percentages (%) were displayed. Analysis of variance (ANOVA) was used for continuous variables and chi-square test was used for categorical variables to test the differences between the groups.

### Observational analysis in the KORA study

Sex-specific correlations of sex hormones were examined by Pearson’s rho. Sex hormone concentrations were sex-specifically z-standardized. The associations between sex hormones and baseline FLI as well as FLI at the follow-up were investigated with linear regression stratified for men and postmenopausal women. Model adjustment was defined *a priori*. The main model was adjusted for age, conventional lifestyle and cardiometabolic risk factors for sex hormone derangement and ectopic fat accumulation, including smoking (never, ex-smoker, smoker), physical activity (active, inactive), alcohol consumption (no intake, moderate intake, excessive intake), systolic blood pressure (SBP), high-density lipoprotein-cholesterol (HDL-C), low-density lilpoprotein-cholesterol (LDL-C) (all continuous), clinically diagnosed diabetes (yes, no), use of antihypertensive medication (yes, no) and lipid lowering medication (yes, no) (**Supplementary Material**). The model for the longitudinal analysis was additionally adjusted for baseline FLI. In a sensitivity analysis, we further adjusted for continuous C-reactive protein (CRP), thyroid stimulating hormone (TSH), serum albumin and SHBG, which are either closely related to sex hormone derangement or determinant for bioavailable sex hormones. The significance level was set to *p*<0.0056 to account for multiple tests (9 exposures) using Bonferroni correction.

### Mendelian randomization analysis

MR analysis was conducted to investigate the causal relationship between sex hormones/SHBG and hepatic PDFF. More detailed explanation of the methodology is provided in the **Supplementary Material**. Firstly, we conducted MR analysis with the inverse-variance weighting (IVW) approach. One of the MR assumptions (exclusion restriction) is that the association between the genetic instrument and the outcome goes only through the exposure (**Supplementary Materials**). The IVW approach is only valid if all genetic instruments fulfill the “exclusion restriction” assumption. In case of genetic pleiotropy where the “exclusion restriction” is violated and genetic variants are also associated with other risk factors of the outcome, other robust MR methods provide valid and consistent MR estimates. The weighted-median approach allows up to 50%, the weighted-mode approach 50% - 100% and the MR-Egger approach up to 100% for pleiotropic variants (31, 32). A statistically significant IVW result with directionally consistent MR estimates from all three sensitivity analyses was considered to be a potential causal effect (33). The existence of directional horizontal pleiotropy was defined if the intercept term of the MR-Egger regression significantly differed from zero (*p* for pleiotropy < 0.05).

For DHEAS, progesterone and 17-OHP, we conducted two-sample MR analysis. Whereas for total T, bioT, E2 and SHBG, MR analysis was carried out in a two-sample setting with population overlap (<10%), since summary statistics for both exposure and outcome were obtained from the UK Biobank. In large scale studies, the precision and bias of MR estimates (except for MR Egger approach) are similar in both two-sample or one-sample (with complete sample overlap) MR settings (34, 35).

In order to investigate the causal effect of T or E2 independent of SHBG, we conducted MR analysis using clusters of genetic instruments with primary effects on specific sex hormone (T or E2 or SHBG) identified previously by Ruth and colleagues (**Supplementary Table 1**) (18). We also conducted sensitivity analysis excluding the SNPs with larger effects on the metabolic risk factors closely related to fatty liver, including fasting glucose, type 2 diabetes, coronary artery disease, HDL-C, LDL-C, triglycerides, total cholesterol, SBP, DBP, BMI and waist-to-hip ratio adjusted for BMI, than their effects on sex hormones identified by Steiger-Filtering previously (18). The Steiger test filters out SNPs that explain more variance in one phenotype (e.g. outcome/trait closely related to the outcome) than another (e.g. exposure), to reduce potential pleiotropic effects of these SNPs and avoid reverse causality (36).

All analyses were conducted using R statistical software, version 4.2.1, including the MR analyses for which we used the “TwoSampleMR” R package. We used multiple imputation with 5 imputed datasets for covariates with missing values less than 5% for the observational analysis. In the MR analysis, a *p*<0.0071 (0.05/7 exposure) was considered significant with Bonferroni correction for multiple testing.

## Results

### Observational analyses

Among 2,239 participants eligible for the cross-sectional analysis, the prevalence of FLI ≥ 60 was higher in men (54%, mean age 57 years) than in postmenopausal women (38%, mean age 66 years). For both men and women, participants with higher FLI were significantly older, had higher BMI, larger waist circumference and they were less physically active. They had higher blood pressure, higher blood lipid concentrations, higher CRP levels and higher liver enzyme levels. They also suffered more from diabetes, and were more likely to use antihypertensive or lipid lowering medication (**Table 1**, **Supplementary Table 2**). Among men, lower levels of sex hormones and SHBG were seen with higher FLI. Whereas among postmenopausal women, higher fT and lower DHEA, DHT and SHBG concentrations were observed with higher FLI (**Table 2**). A correlation matrix between the sex hormones and SHBG is shown in **Supplementary Figure 2**.

**Table 1:** Baseline characteristics of KORA F4 study participants among men and postmenopausal women.

	Men (n=1,456)				Postmenopausal women (n=783)			
	FLI<30 (N=264)	30 ≤ FLI < 60 (N=410)	FLI ≥ 60 (N=782)	*P* value	FLI<30 (N=278)	30 ≤ FLI < 60 (N=208)	FLI ≥ 60 (N=297)	*P* value
**Age (years)**	50.6 (13.0)	56.2 (14.1)	58.8 (12.5)	**< 0.001**	62.7 (8.6)	67.1 (8.2)	66.9 (7.7)	**< 0.001**
**BMI (kg/m^2^)**	23.5 (1.8)	26.0 (1.8)	30.4 (3.9)	**< 0.001**	24.1 (2.4)	27.7 (2.2)	33.3 (4.3)	**< 0.001**
**Waist Circumference (cm)**	86.0 (5.5)	94.6 (5.3)	107.1 (10.5)	**< 0.001**	80.2 (6.2)	90.7 (5.0)	103.7 (9.4)	**< 0.001**
**Smoking**				**< 0.001**				**0.002**
never smoker	105 (39.9%)	132 (32.3%)	212 (27.2%)		150 (54.0%)	137 (65.9%)	186 (62.6%)	
ex-smoker	98 (37.3%)	179 (43.8%)	436 (55.9%)		84 (30.2%)	51 (24.5%)	92 (31.0%)	
smoker	60 (22.8%)	98 (24.0%)	132 (16.9%)		44 (15.8%)	20 (9.6%)	19 (6.4%)	
**Physically active**	164 (62.4%)	243 (59.4%)	371 (47.6%)	**< 0.001**	175 (62.9%)	106 (51.0%)	145 (48.8%)	**0.002**
**Alcohol consumption**				**< 0.001**				**0.049**
no intake	55 (20.9%)	88 (21.5%)	155 (19.9%)		108 (38.8%)	73 (35.1%)	142 (47.8%)	
moderate intake	161 (61.2%)	231 (56.5%)	391 (50.1%)		125 (45.0%)	103 (49.5%)	117 (39.4%)	
excessive intake	47 (17.9%)	90 (22.0%)	234 (30.0%)		45 (16.2%)	32 (15.4%)	38 (12.8%)	
**Systolic blood pressure (mmHg)**	119.8 (15.9)	126.9 (16.9)	131.0 (17.5)	**< 0.001**	119.0 (19.8)	122.7 (19.3)	125.9 (17.3)	**< 0.001**
**Diastolic blood pressure (mmHg)**	73.5 (8.8)	76.4 (9.5)	79.4 (10.4)	**< 0.001**	73.1 (9.3)	73.2 (9.4)	74.1 (9.3)	0.385
**Hypertension**	42 (16.0%)	160 (39.1%)	434 (55.6%)	**< 0.001**	83 (29.9%)	104 (50.0%)	199 (67.2%)	**< 0.001**
**Total cholesterol (mmol/l)**	5.1 (0.9)	5.5 (0.9)	5.6 (1.1)	**< 0.001**	6.0 (0.9)	6.0 (1.0)	6.0 (1.1)	0.758
**HDL-C (mmol/l)**	1.5 (0.3)	1.3 (0.3)	1.2 (0.3)	**< 0.001**	1.8 (0.4)	1.6 (0.3)	1.4 (0.3)	**< 0.001**
**LDL-C (mmol/l)**	3.3 (0.8)	3.6 (0.8)	3.6 (0.9)	**< 0.001**	3.6 (0.9)	3.8 (0.9)	3.8 (0.9)	**0.013**
**Triglycerides (mmol/l)**	0.8 (0.6, 1.1)	1.2 (0.9, 1.5)	1.8 (1.3, 2.5)	**< 0.001**	0.9 (0.7, 1.1)	1.3 (1.0, 1.6)	1.6 (1.2, 2.2)	**< 0.001**
**ALT (ukat/l)**	0.3 (0.3, 0.4)	0.4 (0.3, 0.5)	0.5 (0.4, 0.7)	**< 0.001**	0.3 (0.2, 0.4)	0.3 (0.3, 0.4)	0.4 (0.3, 0.5)	**< 0.001**
**AST (ukat/l)**	0.4 (0.4, 0.5)	0.4 (0.4, 0.5)	0.5 (0.4, 0.6)	**< 0.001**	0.4 (0.3, 0.5)	0.4 (0.3, 0.5)	0.4 (0.3, 0.5)	**< 0.001**
**GGT (U/l)**	24.0 (20.0, 29.0)	31.0 (25.0, 41.0)	46.0 (34.0, 71.8)	**< 0.001**	21.0 (17.0, 26.0)	25.5 (20.0, 36.0)	31.0 (24.0, 48.0)	**< 0.001**
**C-reactive protein (mg/l)**	0.5 (0.3, 1.1)	0.8 (0.5, 1.8)	1.5 (0.8, 2.9)	**0.008**	0.9 (0.5, 1.8)	1.5 (0.9, 3.0)	2.5 (1.4, 4.9)	**< 0.001**
**Diabetes**	5 (2.0%)	43 (10.7%)	147 (19.2%)	**< 0.001**	8 (2.9%)	19 (9.4%)	79 (26.9%)	**< 0.001**
**Antihypertensive medication**	31 (11.8%)	111 (27.1%)	335 (42.8%)	**< 0.001**	74 (26.6%)	95 (45.7%)	182 (61.3%)	**< 0.001**
**Lipid lowering medication**	16 (6.1%)	53 (12.9%)	143 (18.3%)	**< 0.001**	31 (11.2%)	42 (20.2%)	64 (21.5%)	**0.002**
**Thyroid stimulating hormone (mIU/l)**	1.2 (0.8, 1.8)	1.2 (0.9, 1.8)	1.3 (0.9, 1.9)	0.328	1.3 (0.8, 1.9)	1.1 (0.6, 1.7)	1.2 (0.8, 1.7)	0.155
**Serum albumin (g/l)**	45.6 (3.4)	45.3 (3.2)	45.3 (3.5)	0.363	44.0 (3.1)	43.6 (3.0)	43.5 (3.0)	0.102

Values are expressed as the mean (SD) for normally distributed continuous variables or median (interquartile range) for non-normally distributed continuous variables, or n (%) for categorical variables. P-values were generated by ANOVA for continuous variables and chi-square test for categorical variables. P-values< 0.05 are shown in bold.

Excessive alcohol consumption was defined as men with alcohol intake ≥ 30 g/day and women with alcohol intake ≥ 20 g/day.

FLI, fatty liver index; BMI, body mass index; HDL-C, high-density lipoprotein cholesterol; LDL-C, low-density lipoprotein cholesterol; ALT, Alanine Aminotransferase; AST, Aspartate Aminotransferase; GGT, Gamma-Glutamyl Transferase; SD, standard deviation; ANOVA, analysis of variance.

**Table 2:** Baseline characteristics of KORA F4 study participants including sex hormones, SHBG and related variables among men and postmenopausal women.

	Men (n=1,456)				Postmenopausal women (n=783)				Missing
	FLI<30 (N=264)	30 ≤ FLI < 60 (N=410)	FLI ≥ 60 (N=782)	*P* value	FLI<30 (N=278)	30 ≤ FLI < 60 (N=208)	FLI ≥ 60 (N=297)	*P* value	
**Testosterone (nmol/l)**	18.00 (14.47, 21.54)	15.12 (12.06, 19.17)	13.41 (10.36, 16.80)	**< 0.001**	0.66 (0.48, 0.87)	0.58 (0.40, 0.90)	0.62 (0.43, 0.94)	0.325	200 (8.93%)
**Free testosterone (pmol/l)**	208.58 (179.71, 244.65)	194.17 (157.74, 239.79)	183.26 (143.29, 218.49)	**< 0.001**	5.47 (3.54, 8.10)	5.50 (3.92, 8.38)	7.39 (5.12, 10.93)	**< 0.001**	244 (10.90%)
**DHEA (nmol/l)**	10.84 (7.52, 16.04)	9.04 (5.51, 14.55)	7.89 (4.74, 13.13)	**< 0.001**	7.94 (4.58, 11.90)	6.41 (3.93, 9.11)	6.21 (4.17, 9.23)	**< 0.001**	200 (8.93%)
**DHEAS (nmol/l)**	3776.67 (2257.44, 5867.85)	3219.94 (1777.56, 5405.83)	2838.35 (1535.46, 4727.63)	**< 0.001**	1638.94 (940.49, 2429.63)	1267.93 (737.68, 2120.88)	1392.92 (735.54, 2201.20)	0.202	200 (8.93%)
**DHT (nmol/l)**	1.60 (1.17, 2.08)	1.36 (1.04, 1.78)	1.09 (0.76, 1.50)	**< 0.001**	0.21 (0.12, 0.34)	0.17 (0.09, 0.27)	0.15 (0.09, 0.24)	**< 0.001**	200 (8.93%)
**free DHT (pmol/l)**	13.37 (10.47, 17.33)	12.92 (9.72, 16.45)	11.11 (8.43, 14.38)	**< 0.001**	1.33 (0.65, 2.04)	1.15 (0.70, 1.95)	1.27 (0.72, 2.29)	0.399	244 (10.90%)
**Progesterone (nmol/l)**	0.24 (0.15, 0.37)	0.21 (0.11, 0.32)	0.17 (0.09, 0.29)	**< 0.001**	0.12 (0.05, 0.23)	0.12 (0.03, 0.18)	0.09 (0.04, 0.17)	0.569	200 (8.93%)
**17-OHP (nmol/l)**	3.19 (2.38, 4.31)	2.92 (2.29, 3.80)	2.47 (1.78, 3.50)	**< 0.001**	0.80 (0.51, 1.20)	0.79 (0.53, 1.21)	0.79 (0.54, 1.13)	0.545	200 (8.93%)
**SHBG (nmol/l)**	56.00 (41.05, 71.75)	49.45 (37.77, 67.12)	45.70 (31.63, 63.05)	**< 0.001**	88.50 (65.80, 112.55)	74.20 (53.70, 96.80)	53.35 (40.08, 73.65)	**< 0.001**	60 (2.68%)
**Hormone replacement therapy**	NA	NA	NA	NA	26 (9.4%)	11 (5.3%)	15 (5.1%)	0.077	

Values are expressed as the mean (SD) for normally distributed continuous variables or median (interquartile range) for non-normally distributed continuous variables, or n (%) for categorical variables. P-values were generated by ANOVA for continuous variables and chi-square test for categorical variables. P-values< 0.05 are shown in bold.

FLI, fatty liver index; DHEA, dehydroepiandrosterone; DHEAS, dehydroepiandrosterone-sulfate; DHT, dihydrotestosterone; SHBG, sex hormone-binding globulin; 17-OHP, 17α-hydroxyprogesterone; SD, standard deviation; ANOVA, analysis of variance; NA, not applicable.

**Figure 2:**
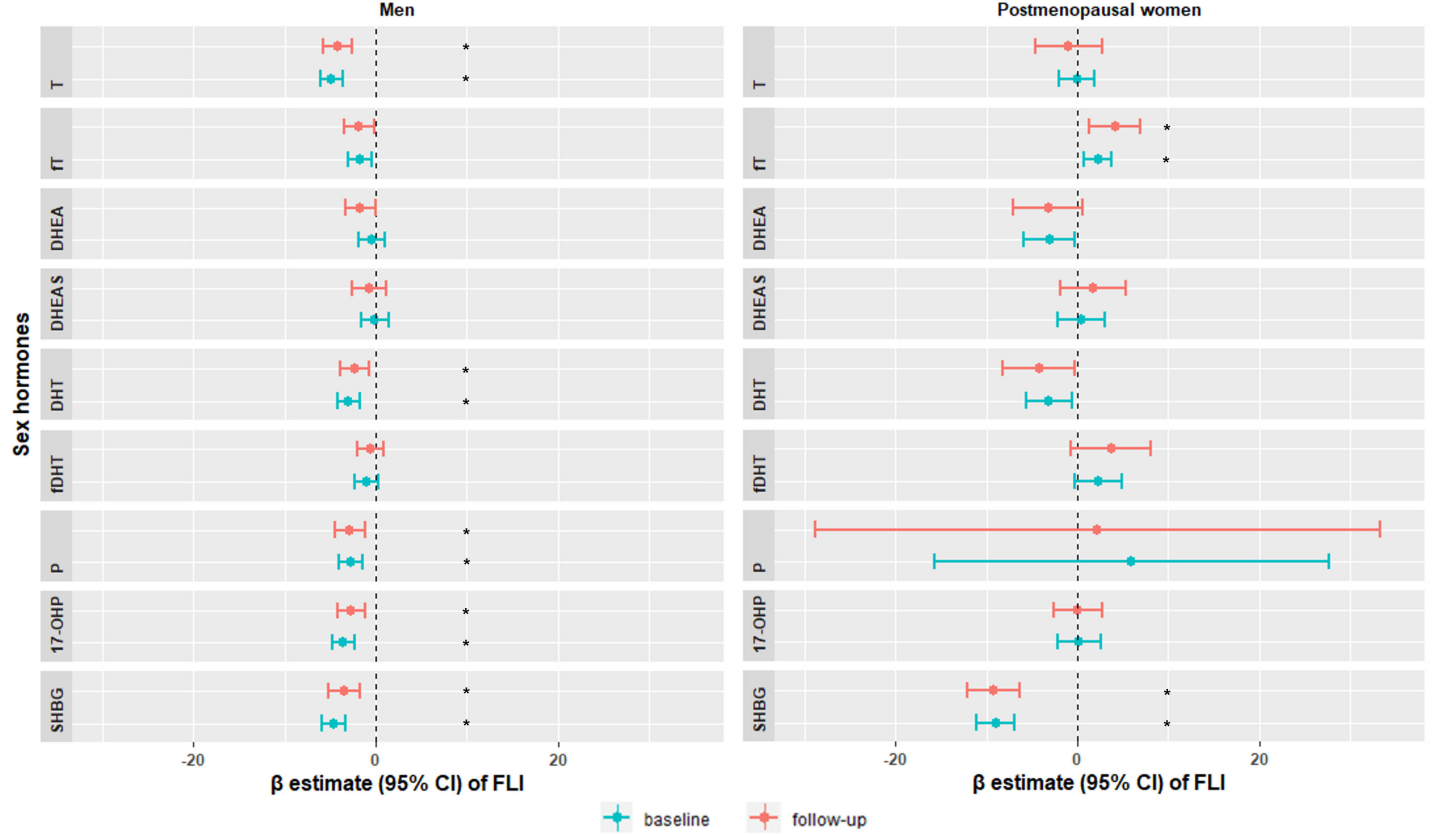
Sex-specific associations of sex hormones with fatty liver index at baseline KORA F4 study (blue) and at follow-up KORA FF4 study (red). Models were adjusted for age, smoking, physical activity, alcohol consumption, SBP, HDL-C, LDL-C, diabetes, antihypertensive medication and lipid lowering medication. Significant associations were labeled with *. T, testosterone; fT, free testosterone; DHEA, dehydroepiandrosterone; DHEAS, dehydroepiandrosterone sulfate; DHT, dihydrotestosterone; fDHT, free dihydrotestosterone; P, progesterone; 17-OHP, 17α-hydroxyprogesterone; SHBG, sex hormone-binding globulin; FLI, fatty liver index; SBP, systolic blood pressure; HDL-C, high-density lipoprotein cholesterol; LDL-C, low-density lipoprotein cholesterol.

Multivariable adjusted regression analyses showed that among men, lower T [β, 95%CI: -4.89 (-6.12, -3.66)], DHT [-2.97 (-4.20, -1.73)], progesterone [-2.75 (-4.02, -1.49)], 17-OHP [-3.57 (-4.80, -2.34)] and SHBG [-4.64 (-5.89, -3.39)] were associated with higher FLI at baseline. Among postmenopausal women, higher fT [2.27 (0.77, 3.77)] and lower SHBG [-9.00 (-11.13, -6.87)] were associated with higher FLI at baseline (**Figure 2** and **Supplementary Table 3**). In longitudinal analysis, similar trends followed for both men and women (**Figure 2** and **Supplementary Table 4**). In the sensitivity analysis, additionally adjusting for CRP, TSH, serum albumin and SHBG hardly changed the associations (**Supplementary Table 5**).

All associations in the longitudinal analysis were attenuated after adjustment for baseline FLI (**Supplementary Table 4**), possibly due to reverse causation. However, baseline adjustment is only occasionally advantageous, and whether it eliminates or introduces bias depends crucially upon the causal structure relating the variables (37).

### Mendelian randomization analysis

For sex hormones, the MR IVW estimates for total T [-0.09 (-0.16, -0.01)] in men and bioavailable T [0.13 (0.03, 0.23)] in women were nominally significant (*p*<0.05), but they did not pass the significance level of *p*< 0.0071 after Bonferroni correction. MR analyses with the IVW approach revealed that higher SHBG among women [-0.36 (-0.61, -0.12)] was associated with lower hepatic PDFF. Among men, the estimate was smaller [-0.19 (-0.33, -0.05)], and did not pass the Bonferroni threshold. Sensitivity analyses with weighted median, weighted mode and MR-Egger yielded estimates directionally consistent to the IVW estimates (**Table 3**). There was no indication of directional horizontal pleiotropy in the above MR analyses (*p* for pleiotropy from MR-Egger ≥ 0.05) (**Table 3**).

**Table 3:** Mendelian randomization estimates of the relationship between sex hormones/SHBG on hepatic proton density fat fraction.

Exposure	Sex	N instruments	IVW		Weighted median		Weighted mode		MR-Egger		
			β (95% CI)	*P value*	β (95% CI)	*P value*	β (95% CI)	*P value*	β (95% CI)	*P value*	*P for* pleiotropy
**Total testosterone**	Men	104	-0.09 (-0.16, -0.01)	0.020	-0.05 (-0.13, 0.02)	0.157	-0.03 (-0.09, 0.03)	0.343	-0.02 (-0.14, 0.10)	0.713	0.163
	Women	124	-0.05 (-0.11, 0.01)	0.121	0.02 (-0.05, 0.09)	0.529	0.01 (-0.06, 0.08)	0.800	0.004 (-0.10, 0.11)	0.943	0.229
**Bioavailable testosterone**	Men	57	0.003 (-0.06, 0.06)	0.927	0.02 (-0.09, 0.12)	0.733	0.02 (-0.09, 0.13)	0.677	0.04 (-0.07, 0.14)	0.496	0.448
	Women	88	0.13 (0.03, 0.23)	0.012	0.13 (0.02, 0.24)	0.016	0.11 (0.01, 0.22)	0.036	0.10 (-0.09, 0.28)	0.312	0.678
**Estradiol**	Men	10	-0.35 (-1.16, 0.47)	0.400	-0.03 (-0.74, 0.68)	0.935	0.08 (-0.61, 0.77)	0.823	1.75 (-0.19, 3.69)	0.115	0.053
**Progesterone**	Women	3	0.004 (-0.06, 0.07)	0.910	-0.02 (-0.09, 0.05)	0.563	-0.03 (-0.11, 0.05)	0.531	0.19 (-0.48, 0.87)	0.678	0.681
**17-OHP**	Men	4	0.10 (-0.0003, 0.20)	0.051	0.04 (-0.04, 0.12)	0.290	0.05 (-0.04, 0.13)	0.351	0.01 (-0.18, 0.20)	0.912	0.404
	Women	2	0.01 (-0.01, 0.03)	0.247	NA	NA	NA	NA	NA	NA	NA
**DHEAS**	Sex-combined	4	0.01 (-0.16, 0.18)	0.916	0.03 (-0.11, 0.16)	0.694	0.07 (-0.05, 0.20)	0.341	0.26 (0.05, 0.47)	0.141	0.119
**SHBG**	Men	151	-0.19 (-0.33, -0.05)	0.0074	-0.09 (-0.22, 0.03)	0.151	-0.04 (-0.15, 0.06)	0.422	-0.14 (-0.34, 0.07)	0.190	0.479
	Women	160	**-0.36 (-0.61, -0.12)**	**0.004**	-0.18 (-0.32, -0.05)	0.008	-0.16 (-0.29, -0.02)	0.023	-0.14 (-0.53, 0.26)	0.503	0.150

Mendelian randomization analysis was carried out with the inverse-variance weighted approach as the main analysis, and robust methods such as weighted median, weighted mode and MR-Egger were carried out as sensitivity analyses. The robust methods allow for certain percentage of invalid (e.g. pleiotropic) instrumental SNPs in the Mendelian randomization analysis, and provide estimates of causal effect not subject to these violations. A statistically significant IVW result with directionally consistent Mendelian randomization estimates from all three sensitivity analyses was considered to be a potential causal effect.

P for pleiotropy is the p value to reject the null hypothesis that the intercept term of the MR Egger regression equals to zero. P for pleiotropy < 0.05 indicates the existence of directional pleiotropy.

P<0.0071 (0.05/7) is considered significant with Bonferroni correction for multiple testing and was shown in bold.

SHBG, sex hormone-binding globulin; DHEAS, Dehydroepiandrosterone-sulfate; 17-OHP, 17α-hydroxyprogesterone; IVW, inverse-variance weighted; NA, Not applicable.

Due to genetic overlap between T, E2 and SHBG, we used clusters of instrumental SNPs with primary effects on T or E2 or SHBG to investigate the potential causal effect of T or E2 on hepatic PDFF independent of SHBG. MR analysis with clusters of T or E2 showed that there was no association between T and hepatic PDFF independent of SHBG in either men or women. Nor was there any association between E2 and hepatic PDFF independent of SHBG in men (**Supplementary Table 6**). The IVW estimates for both male SHBG cluster [-0.20 (-0.34, -0.06)] and female SHBG cluster [-0.43 (-0.61, -0.25)] reached statistical significance after Bonferroni correction. All three sensitivity analyses resulted in estimates in the same direction as the IVW estimates (**Supplementary Table 6**). The male SHBG cluster includes SNPs with primary SHBG increasing effect and secondary increasing effect on total T and decreasing effect on bioT as well as increasing effect on E2, and the female SHBG cluster includes SNPs with primary increasing effect on SHBG and secondary opposing effect on T and bioT. Taken together, this indicated that genetically determined higher SHBG has a decreasing effect on hepatic PDFF in both men and women, probably also through its effect on sex hormones (**Table 3**).

In order to minimize the pleiotropic effect of SNPs closely associated with metabolic risk factors, we further excluded them from the MR. The association between SHBG and hepatic PDFF attenuated, but maintained the same directionality (**Supplementary Table 7**).

## Discussion

In this study, we investigated the observational and possible causal association of endogenous sex hormones and SHBG with liver fat combining evidence from a population-based study and summary-level data from the largest up to date GWAS. We observed that higher sex hormones, such as T, DHT, progesterone, 17-OHP, as well as SHBG were associated with lower FLI both at baseline and follow-up among men. Among postmenopausal women, lower fT and higher SHBG were both associated with lower FLI at baseline and follow-up. The MR analyses showed suggestive evidence for an inverse causal association of genetically determined SHBG on hepatic fat content in women, but no other potential causal effect was found for sex hormones on liver fat.

A recent meta-analysis including 16 studies found that higher T was associated with lower odds of fatty liver [0.59 (0.42, 0.76)] in men, but not in women. In KORA, we confirmed these results. Interestingly, although we did not find an association between T and FLI among women, higher fT was associated with higher FLI both cross-sectionally and longitudinally. Previous epidemiological evidence also suggested similar associations between fT or bioT levels and higher risk of fatty liver in women (38, 39). This indicates that not the total amount of circulating T but rather the amount of directly available T to the tissues is strongly related with fatty liver risk, especially in women. This could also be a secondary effect of SHBG, whose increase can reduce the levels of fT.

In a clinical trial, obese men treated with T had substantially increased muscle mass and improved insulin sensitivity as well as reduced liver fat, possibly owing to the protective role of T to regulate body composition and glucose metabolism in men (3, 40). However, T seems to exert a distinct metabolic effect in women, potentially due to decreased conversion of T to E2. Additionally, postmenopausal women are be at higher risk of fatty liver, as a result of weight gain, lipid dysregulation and unfavorable adipose distribution due to declining E2 levels (2, 10). In alignment, we found that fT was associated with FLI in opposite ways for men (inversely) and women (positively) in our study.

Although lower DHEAS levels were observed in the group of biopsy-proven more advanced fatty liver disease involving inflammation and fibrosis in a small study (8), we did not find any association between DHEA or DHEAS with FLI in our study sample. Our finding was supported by the null association in a population-based study comparing the risk of ultrasound diagnosed fatty liver in relation to DHEA and DHEAS levels (4). Our analysis also suggested inverse associations of DHT, progesterone and 17-OHP with FLI in men. Experimental studies have shown that DHT, progesterone and 17-OHP influence lipid and glucose metabolism and regulate inflammatory proteins, such as by interacting with insulin signaling in adipocytes or activating glucocorticoid receptor in the liver (12, 41, 42). However, there isn’t yet consistent evidence from population-based studies linking these sex hormones to fatty liver. Further studies are needed to examine the role of these sex hormones and fatty liver risk longitudinally.

We noted that lower SHBG levels were associated with higher FLI in both men and women, which is consistent with the findings from a recent meta-analysis (4). Previous literature has shown that lower endogenous SHBG level is associated with higher risk of cardiometabolic disorders and fatty liver, and this association is reported to be constant in both sexes across age groups (4, 43, 44). Moreover, lower SHBG has been associated with older age, obesity, and lifestyle risk factors, such as being physically inactive and alcohol consumption, all closely related to liver fat accumulation (45, 46). In our study, the association between SHBG and FLI remained significant after adjusting for all these factors. However, given the multifactorial nature of fatty liver, there might be other risk factors confounding the observed associations, which we were not able to correct for. Although the mechanism underlying the association between SHBG and liver fat regulation remains uncertain, animal experiments implied that increased SHBG level can downregulate the expression of the crucial enzymes involved in the hepatic lipogenesis, such as the adenosine triphosphate (ATP) citrate lyase (production of precursor for fatty acid), Acetyl-CoA-carboxylase and fatty acid synthase (further restriction of fatty acid synthesis) in the liver (47, 48), which could consequently reduce liver fat content. Meanwhile, *in vitro* experiments showed that SHBG can repress inflammatory cytokines, including interleukin-6 and tumor necrosis factor-alpha in adipocztes and macrophages, modulating inflammatory processes (48). Furthermore, SHBG may indirectly impact liver fat content by regulating the bioavailability and balance of sex hormones. On the other hand, liver cell function and other metabolic factors, such as insulin, can also regulate SHBG production (13). Additionally, the genetic determinants of SHBG overlap with those of other metabolic risk factors for fatty liver, as captured in our MR analysis. Therefore, the observational association between SHBG and risk of liver fat accumulation could be subject to residual confounding and reverse causation, which can be better addressed with MR analysis.

Previous MR studies have suggested the protective role of SHBG against the development of metabolic disorders, such as type 2 diabetes (18) and hypertension (49), both risk factors for fatty liver. Accordingly, we found that genetically determined circulating SHBG were inversely associated with liver fat content in women, consistent with the observational evidence. However, among men, this association was only nominal (*p*<0.05) but did not pass the Bonferroni correction threshold of *p*<0.0071. Although we did not detect any pleiotropy using a battery of robust MR methods, the associations between SHBG and hepatic PDFF should be interpreted with caution, since the association was attenuated after we excluded SNPs closely related to metabolic risk factors identified by Steiger-filtering in a previous study (18). This finding highlights the importance of carefully evaluating the assumptions underlying the MR analysis and employing appropriate methods to address potential confounding effects of metabolic risk factors, highly intermingled in fatty liver pathophysiology. We did not find implication regarding potential causal effect of sex hormones on liver fat.

This is the first study investigating the sex-specific role of a wide range of sex hormones in liver fat accumulation with both observational evidence from a well-characterized population-based study as well as genetic data. Sex hormones were quantified by mass spectrometry, increasingly recognized as the gold standard, being more accurate and sensitive compared to the widely-used immunoassay (50). Using multiple genetic instruments and several MR sensitivity analyses, we could address the potential existence of horizontal pleiotropy and the robustness of the MR estimates. Nevertheless, our study also entails several limitations. We were unable to quantify the role of E2 in relation to liver fat. Although there is evidence indicating a protective role of E2 on liver injury and liver fat accumulation (2, 51), epidemiological studies comparing the risk of fatty liver related to the endogenous levels of E2 could not find a significant association between these two (4). Meanwhile, even though the administration of exogenous E2 has been shown to be associated with an increase in SHBG levels (52), we expect the effect of circulating E2 on SHBG to be neglectable in our study population of postmenopausal women and men since E2 levels are low and stable in this group (53). Nevertheless, future studies should focus on determining the impact of endogenous E2 levels and liver fat and, in particular, addressing the challenge of periodic fluctuations in E2 in premenopausal women. MRI has been deemed to be the gold standard for non-invasive measurement for liver fat content, but the high cost of MRI precludes it for large scale investigations. We did not use sex-specific genetic associations with hepatic PDFF for the MR analysis, but we don’t expect large differences - a GWAS from the UK Biobank indicated no sex difference in the genetic signals for steatohepatitis (29). Up to date, the GWAS from UK Biobank include the highest number of genetic instruments for T, E2 and SHBG. Therefore, we employed the two-sample approach with sample overlap (<10%) for these exposures, which could bias the MR estimates towards the observational associations (weak instrument bias). However, in case of large study population, using strong genetic instruments (*p* < 5e-8) which explain high genetic heritability of the phenotypes (2% -21%), potential bias due to weak instruments is expected to be low (35).

## Conclusion

Our complementary observational and MR results support suggestive causal associations between SHBG with liver fat, particularly in women, indicating that interventions targeting this pathway, along with management of accompanying risk factors, may help the prevention of fatty liver. Further observational studies are needed to examine the sex-specific associations between sex hormones and liver fat accumulation quantified by MRI using population-based data.

## Data availability statement

The data underlying this article cannot be shared publicly due to data protection reasons. The data will be shared on reasonable request to the corresponding author.

## Ethics statement

The studies involving humans were approved by the Ethics Committees of the Bavarian Chamber of Physicians (Ethical Approval Number 06068). The studies were conducted in accordance with the local legislation and institutional requirements. The participants provided their written informed consent to participate in this study.

## Author contributions

All persons listed as coauthors have significantly contributed to preparing the manuscript: XC has designed the analyses, interpreted the data and drafted the manuscript; JN and BT have contributed to the conception, design and interpretation of the data and approval of the manuscript; SH, CP, AC, JA, TZ, AD, RB, AP, HY have contributed substantially to the interpretation of the data and have critically revised the manuscript for important intellectual content. All authors contributed to the article and approved the submitted version.
