# Supplementary material for: Association of sex hormones and sex hormone-binding globulin with liver fat in men and women: an observational and Mendelian randomization study
Source: Front Endocrinol (Lausanne). 2023 Oct 13;14:1223162. doi: 10.3389/fendo.2023.1223162 (PMC10611498; doi:10.3389/fendo.2023.1223162)
Supplement: Supplementary file 1 [file DataSheet_1.docx]

**Supplementary Materials**

**Methods**

**Laboratory and clinical measurements**

A blood sample without stasis was taken from the participants after an overnight fast of at least 8 hours and kept at 4°C until it was centrifuged. The Roche/Hitachi Cobas® system (Roche, Mannheim, Germany) was used to analyze liver enzymes GGT, aspartate aminotransferase (AST) and alanine aminotransferase (ALT) according to the recommendations of the International Federation of Clinical Chemistry from 1983 (confirmed and extended in 2002) (1). Serum total cholesterol (CHOL Flex), high-density lipoprotein cholesterol (HDL-C) (AHDL Flex), low-density lipoprotein cholesterol (LDL-C) (ALDL Flex) concentrations were measured by the enzymatic methods (CHOD-PAP; Dade Behring, Marburg, Germany). Triglycerides (TG) were measured by an enzymatic color test (GPO-PAP method, TGL Flex; Dade Behring, Marburg, Germany). High-sensitivity C-reactive protein (CRP), thyroid stimulation hormone (TSH), sex hormone-binding globulin (SHBG) and serum albumin were determined from the frozen plasma and serum samples which were stored at -80 °C until assayed. CRP was determined by nephelometry on a BN II analyzer (Siemens, Erlangen, Germany). TSH was measured by the immunochemiluminescent procedure (Dimension Vista System, Siemens). Serum SHBG was measured by a chemiluminiscent microparticle immunoassay, the ARCHITECT SHBG assay (Abbott Diagnostics). Serum albumin was measured by immunonephelometry (ALB Flex; Dade Behring, Germany). Self-reported type 1 diabetes, type 2 diabetes, or medication-induced diabetes cases were validated by contacting the individual’s general practitioners or medical chart review. Among subjects without known diabetes diagnosis, an oral glucose tolerance test (OGTT) was performed. Diabetes (fasting glucose > 6.9 mmol/L and/or 2h glucose > 11.0 mmol/L) was defined according to the 1999 World Health Organization diagnostic criteria (2). Diabetes (yes, no) was defined if the participant self-reported any type of diabetes or was newly diagnosed with diabetes by OGTT.

**Anthropometric measurements and interviews**

Waist circumference, height, weight, systolic and diastolic blood pressure (SBP and DBP) were measured based on protocols described elsewhere (3). Body mass index (BMI) was calculated as weight (kg) divided by height squared (m^2^). Waist circumference (cm) was measured at the level midway between the lower rib margin and the iliac crest while the participants breathed out gently. Baseline information on sociodemographic status, medical history, medication use, physical activity level, alcohol consumption as well as smoking habit were ascertained during an interview given by trained medical workers (1).

Alcohol consumption (g/day) calculated from beer, wine and spirits intake on weekday and weekend was categorized as follows: no alcohol consumption, moderate alcohol consumption (men: >0 and <30 g/day; women: >0 and <20 g/day), and excessive alcohol consumption (men: ≥ 30 g/day; women: ≥ 20 g/day) (4). The duration of leisure time sport activity in winter and summer was assessed separately as follows: >2h/week (scored 1), 1-2 h/week (scored 2), <1 h/week (scored 3), none (scored 4). Possible scores for summer and winter were summed up to generate a total score for physical activity. Participants were classified as ‘physically inactive’ if they had a total score ≥ 5, and ‘physically active’ otherwise. Smoking status was classified as never smoker, ex-smoker and smoker. Hormone replacement therapy included use of hormonal medications containing natural or synthetic estrogen. Medication use within the last seven days prior to the interview, such as use of antihypertensive and lipid-lowering medication, was ascertained (5).

**Mendelian Randomization analysis**

**Introduction of the method**

In traditional epidemiological studies, causal inference is hampered by the possibility of residual confounding and reverse causality. Mendelian randomization (MR) can be used to identify causal relationships, where genetic variants are used as proxies for the exposure of interest, as Mendel's laws of inheritance indicate that alleles segregate randomly from parents to offspring. Moreover, the germ-line genotypes are fixed at conception and thus offspring genes are unlikely to be associated with confounding factors under observation. Therefore, using genetic variants to represent potentially modifiable risk factors, MR could avoid issues of residual confounding and reverse causation in the observational studies. However, it is important to note that a person’s genetics influence their biology from conception, meaning causal estimates from MR studies may reflect the effect of life-long exposure on the outcome of interest (6). Although in the case of multifactorial diseases such as fatty liver, a single genetic variant cannot explain disease occurrence, the emerging genome wide associations studies with ever increasing sample sizes, have provided a tremendous opportunities to investigate genetic determinants of complex diseases in well-powered settings.

**Assumptions**

There are three assumptions for MR analysis, including relevance (strong and stable causal link between the genetic variants and the exposure), independence (absence of confounders in the association between the genetic variants and outcome), and exclusion restriction (no association between the genetic variants and outcome other than through the exposure) (7).

**Further exploratory MR analysis in our study**

In order to investigate the causal effect of T or E2 independent of SHBG, we also conducted MR analysis using clusters of genetic instruments with primary effects on specific sex hormone (T or E2 or SHBG) identified previously by Ruth and colleagues (8). These included: (1) a male T cluster with primary increasing effect on T and secondary increasing effect on E2 but independent of SHBG; (2) a male SHBG cluster with primary SHBG increasing effect and secondary increasing effect on total T and decreasing effect on bioT as well as increasing effect on E2; (3) a male E2 cluster with only increasing effect on E2; (4) a female T cluster with primary T increasing effect independent of SHBG; (5) a female SHBG cluster with primary increasing effect on SHBG and secondary opposing effect on T and bioT (**Supplementary Table 1**) (8).

**References**

1. Rückert I-M, Heier M, Rathmann W, Baumeister SE, Döring A, Meisinger C. Association between markers of fatty liver disease and impaired glucose regulation in men and women from the general population: the KORA-F4-study. PLoS One. 2011;6(8):e22932.

2. Organization WH. Definition, diagnosis and classification of diabetes mellitus and its complications: report of a WHO consultation. Part 1, Diagnosis and classification of diabetes mellitus. Geneva: World health organization; 1999.

3. Rathmann W, Haastert B, Icks Aa, Löwel H, Meisinger C, Holle R, et al. High prevalence of undiagnosed diabetes mellitus in Southern Germany: target populations for efficient screening. The KORA survey 2000. Diabetologia. 2003;46(2):182-9.

4. EASL, EASD, EASO. EASL-EASD-EASO Clinical Practice Guidelines for the management of non-alcoholic fatty liver disease. J Hepatol. 2016;9(2):65-90.

5. Scholze J. Empfehlungen zur Hochdruckbehandlung in der Praxis. Notfall & Hausarztmedizin. 2005;31(04):152-9.

6. Burgess S, Thompson SG. Mendelian randomization: methods for causal inference using genetic variants: CRC Press; 2021.

7. Davies NM, Holmes MV, Davey Smith G. Reading Mendelian randomisation studies: a guide, glossary, and checklist for clinicians. BMJ. 2018;362:k601.

8. Ruth KS, Day FR, Tyrrell J, Thompson DJ, Wood AR, Mahajan A, et al. Using human genetics to understand the disease impacts of testosterone in men and women. Nat Med. 2020;26(2):252-8.

| **Supplementary Table 1. Genome-wide association studies included for the Mendelian Randomization analyses.** | | | | | | | |
| --- | --- | --- | --- | --- | --- | --- | --- |
| **Sex hormone (Exposure)** | **N instruments identified in the original study** | **N instruments after exclusion*** | **Sample size** | **Accession ID in GWAS Catalogue** | **Study** | **First author** | **Cohort** |
| **Testosterone (men)** | **231** | **104** | **178,782** | GCST90012113 | Using human genetics to understand the disease impacts of testosterone in men and women. (PMID: 32042192) | Ruth KS ***2020*** | UK Biobank |
| **(Male testosterone cluster)** | **(122)** | **51** |  |  |  |  |  |
| **Testosterone (women)** | **254** | **124** | **230,454** | GCST90012112 |  |  |  |
| **(Female testosterone cluster)** | **(241)** | **116** |  |  |  |  |  |
| **Bioavailable T (men)** | **125** | **57** | **194,453** | GCST90012103 |  |  |  |
| **Bioavailable T (women)** | **180** | **88** | **188,507** | GCST90012102 |  |  |  |
| **Estradiol (men)** | **22** | **10** | **206,927** | GCST90012105 |  |  |  |
| **(Male estradiol cluster)** | **(14)** | **8** |  |  |  |  |  |
| **SHBG (men)** | **357** | **151** | **180,094** | GCST90012108 |  |  |  |
| **(Male SHBG cluster)** | **(362)** | **148** |  |  |  |  |  |
| **SHBG (women)** | **359** | **160** | **188,908** | GCST90012106 |  |  |  |
| **(Female SHBG cluster)** | **(373)** | **159** |  |  |  |  |  |
| **DHEAS (sex-combined)** | **8** | **4** | **14,846** | GCST001038 | Eight common genetic variants associated with serum DHEAS levels suggest a key role in ageing mechanisms. (PMID: 21533175) | Zhai G ***2011*** | TwinsUK, Rotterdam Study 1, inCHIANTI, Health ABC, GOOD, SHIP |
| **Progesterone (men)** | **22** | **0** | **2,220** | GCST90094672 | Sex-Specific Causal Relations between Steroid Hormones and Obesity-A Mendelian Randomization Study. (PMID: 34822396) | Pott J ***2021*** | LIFE-Heart and LIFE-Adult (Leipzig) |
| **Progesterone (women)** | **5** | **3** | **1,877** | GCST90094673 |  |  |  |
| **17-OHP (men)** | **67** | **4** | **2,220** | GCST90094675 |  |  |  |
| **17-OHP (women)** | **28** | **2** | **1,329** | GCST90094676 |  |  |  |
| **(Outcome)** |  |  | **Sample size** |  | **Study** | **First author** | **Cohort** |
| **Hepatic MRI-PDFF** | **-** | **-** | **14,440** | - | Genome-wide and Mendelian randomisation studies of liver MRI yield insights into the pathogenesis of steatohepatitis. (PMID: 32247823) | Parisinos C ***2020*** | UK Biobank |
| *Abbreviations:* SHBG, sex hormone-binding globulin; DHEAS, dehydroepiandrosterone-sulfate; GWAS, genome-wide association study; 17-OHP, 17α-hydroxyprogesterone; MRI-PDFF, magnetic resonance imaging-proton density fat fraction; SNP, single-nucleotide polymorphism.  * The number of genetic instruments after clumping, harmonization and dropping palindromic SNPs. | | | | | | | |

| **Supplementary Table 2. Baseline characteristics of KORA F4 study participants.** | | |
| --- | --- | --- |
|  | **Men (N=1,456)** | **Postmenopausal women (N=783)** |
| **Age (years)** | 56.6 (13.4) | 65.5 (8.4) |
| **BMI (kg/m^2^)** | 27.9 (4.2) | 28.5 (5.1) |
| **Waist Circumference (cm)** | 99.8 (12.0) | 91.9 (12.5) |
| **Smoking** |  |  |
| never smoker | 449 (30.9%) | 473 (60.4%) |
| ex-smoker | 713 (49.1%) | 227 (29.0%) |
| current smoker | 290 (20.0%) | 83 (10.6%) |
| **Physically active** | 778 (53.6%) | 426 (54.4%) |
| **Alcohol consumption** |  |  |
| no intake | 298 (20.5%) | 323 (41.3%) |
| moderate intake | 783 (53.9%) | 345 (44.1%) |
| excessive intake | 371 (25.6%) | 115 (14.7%) |
| **Systolic blood pressure (mmHg)** | 127.8 (17.5) | 122.6 (19.0) |
| **Diastolic blood pressure (mmHg)** | 77.5 (10.1) | 73.5 (9.3) |
| **Hypertension** | 636 (43.8%) | 386 (49.4%) |
| **Total cholesterol (mmol/l)** | 5.5 (1.0) | 6.0 (1.0) |
| **HDL (mmol/l)** | 1.3 (0.3) | 1.6 (0.4) |
| **LDL (mmol/l)** | 3.6 (0.9) | 3.7 (0.9) |
| **Triglycerides (mmol/l)** | 1.3 (0.9, 1.9) | 1.2 (0.9, 1.7) |
| **ALT (ukat/l)** | 0.4 (0.3, 0.6) | 0.3 (0.3, 0.4) |
| **AST (ukat/l)** | 0.4 (0.4, 0.5) | 0.4 (0.3, 0.5) |
| **GGT (U/l)** | 35.0 (26.0, 53.0) | 25.0 (20.0, 36.0) |
| **C-reactive protein (mg/l)** | 1.1 (0.6, 2.4) | 1.6 (0.8, 3.2) |
| **Diabetes** | 195 (13.7%) | 106 (13.8%) |
| **Antihypertensive medication** | 477 (32.8%) | 351 (44.8%) |
| **Lipid lowering medication** | 212 (14.6%) | 137 (17.5%) |
| **Testosterone (nmol/l)** | 14.6 (11.4, 18.7) | 0.6 (0.4, 0.9) |
| **Free T (pmol/l)** | 191.3 (151.9, 229.3) | 6.1 (4.1, 9.5) |
| **DHEA (nmol/l)** | 8.8 (5.4, 14.3) | 6.8 (4.2, 10.2) |
| **DHEAS (nmol/l)** | 3149.2 (1718.6, 5219.1) | 1426.1 (786.2, 2257.7) |
| **DHT (nmol/l)** | 1.2 (0.9, 1.7) | 0.2 (0.1, 0.3) |
| **free DHT (pmol/l)** | 12.0 (9.0, 15.5) | 1.3 (0.7, 2.1) |
| **Progesterone (nmol/l)** | 0.2 (0.1, 0.3) | 0.1 (0.00, 0.2) |
| **17-OHP (nmol/l)** | 2.7 (2.0, 3.7) | 0.8 (0.5, 1.2) |
| **SHBG (nmol/l)** | 48.3 (35.2, 65.6) | 71.0 (49.7, 98.9) |
| **Thyroid stimulating hormone (mIU/l)** | 1.3 (0.9, 1.8) | 1.2 (0.7, 1.8) |
| **Serum albumin (g/l)** | 45.3 (3.4) | 43.7 (3.0) |
| **Hormone replacement therapy** | NA | 52 (6.6%) |
| Values are expressed as the mean (SD) for normally distributed continuous variables or median (interquartile range) for non-normally distributed continuous variables, or n (%) for categorical variables.  Excessive alcohol consumption was defined as men with alcohol intake ≥ 30 g/day and women with alcohol intake ≥ 20 g/day.  *Abbreviations:* FLI, fatty liver index; BMI, body mass index; HDL-C, high-density lipoprotein cholesterol; LDL-C, low-density lipoprotein cholesterol; ALT, Alanine Aminotransferase; AST, Aspartate Aminotransferase; GGT, Gamma-Glutamyl Transferase; DHEA, dehydroepiandrosterone; DHEAS, dehydroepiandrosterone-sulfate; DHT, dihydrotestosterone; SHBG, sex hormone-binding globulin; 17-OHP, 17α-hydroxyprogesterone; SD, standard deviation. | | |

| **Supplementary Table 3. Association of endogenous sex hormones and SHBG with fatty liver index (continuous) in the baseline KORA F4 study.** | | | | | | |
| --- | --- | --- | --- | --- | --- | --- |
|  | **Men** | | | **Postmenopausal women** | | |
|  | N | **β, 95% CI** | *P value* | N | **β, 95% CI** | *P value* |
| **Testosterone** | 1,358 | **-4.89 (-6.12, -3.66)** | **<0.001** | 681 | -0.05 (-2.05, 1.94) | 0.957 |
| **Free testosterone** | 1,328 | -1.76 (-3.06, -0.46) | 0.008 | 667 | **2.27 (0.77, 3.77)** | **0.003** |
| **DHEA** | 1,358 | -0.44 (-1.89, 1.01) | 0.549 | 681 | -3.05 (-5.87, -0.23) | 0.035 |
| **DHEAS** | 1,358 | -0.10 (-1.60, 1.39) | 0.893 | 681 | 0.46 (-2.11, 3.03) | 0.729 |
| **DHT** | 1,358 | **-2.97 (-4.20, -1.73)** | **<0.001** | 681 | -3.14 (-5.66, -0.62) | 0.015 |
| **Free DHT** | 1,328 | -1.03 (-2.29, 0.22) | 0.106 | 667 | 2.24 (-0.36, 4.83) | 0.092 |
| **Progesterone** | 1,358 | **-2.75 (-4.02, -1.49)** | **<0.001** | 681 | 5.97 (-15.72, 27.65) | 0.590 |
| **17-OHP** | 1,358 | **-3.57 (-4.80, -2.34)** | **<0.001** | 681 | 0.17 (-2.18, 2.53) | 0.886 |
| **SHBG** | 1,417 | **-4.64 (-5.89, -3.39)** | **<0.001** | 762 | **-9.00 (-11.13, -6.87)** | **<0.001** |
| Model was adjusted for age, smoking, physical activity, alcohol consumption, SBP, HDL-C, LDL-C, diabetes, antihypertensive medication and lipid lowering medication. | | | | | | |
| Sex hormones were sex-specifically z-standardized prior to the analysis. The coefficient estimates represent the change of the outcome corresponding to 1-standard deviation increase of the sex hormone.  P<0.0056 (0.05/9) is considered significant with Bonferroni correction for multiple testing.  *Abbreviations*: SBP, systolic blood pressure; HDL-C, high-density lipoprotein cholesterol; LDL-C, low-density lipoprotein cholesterol; DHEA, dehydroepiandrosterone; DHEAS, dehydroepiandrosterone-sulfate; DHT, dihydrotestosterone; SHBG, sex hormone-binding globulin; 17-OHP, 17α-hydroxyprogesterone; β, β-estimate. | | | | | | |

| **Supplementary Table 4. Association of endogenous sex hormones and SHBG with fatty liver index in the follow-up KORA FF4 study.** | | | | | | |
| --- | --- | --- | --- | --- | --- | --- |
|  | **Men** | | | **Postmenopausal women** | | |
| **Model adjusted for age, lifestyle- and metabolic risk factors** | | | | | | |
|  | N | **β, 95% CI** | *P value* | **N** | **β, 95% CI** | *P value* |
| **Testosterone** | 959 | **-4.23 (-5.83, -2.62)** | **<0.001** | 416 | -0.95 (-4.61, 2.71) | 0.610 |
| **Free testosterone** | 941 | -1.81 (-3.44, -0.19) | 0.029 | 408 | **4.17 (1.35, 6.98)** | **0.004** |
| **DHEA** | 959 | -1.67 (-3.39, 0.04) | 0.056 | 416 | -3.24 (-7.11, 0.63) | 0.101 |
| **DHEAS** | 959 | -0.75 (-2.60, 1.11) | 0.431 | 416 | 1.76 (-1.81, 5.32) | 0.334 |
| **DHT** | 959 | **-2.30 (-3.82, -0.79)** | **0.003** | 416 | -4.23 (-8.18, -0.28) | 0.037 |
| **Free DHT** | 941 | -0.59 (-2.08, 0.90) | 0.439 | 408 | 3.72 (-0.67, 8.11) | 0.098 |
| **Progesterone** | 959 | **-2.85 (-4.47, -1.22)** | **0.001** | 416 | 2.23 (-28.86, 33.32) | 0.888 |
| **17-OHP** | 959 | **-2.68 (-4.20, -1.16)** | **0.001** | 416 | 0.03 (-2.64, 2.70) | 0.982 |
| **SHBG** | 1003 | **-3.45 (-5.13, -1.78)** | **<0.001** | 468 | **-9.23 (-12.19, -6.28)** | **< 0.001** |
| **Model + baseline FLI** | | | | | | |
| **Testosterone** | 959 | 0.60 (-0.45, 1.65) | 0.265 | 416 | -0.41 (-2.25, 1.42) | 0.659 |
| **Free testosterone** | 941 | 0.14 (-0.89, 1.17) | 0.790 | 408 | -0.15 (-1.60, 1.29) | 0.835 |
| **DHEA** | 959 | -0.79 (-1.87, 0.29) | 0.151 | 416 | -0.79 (-2.73, 1.16) | 0.427 |
| **DHEAS** | 959 | -0.48 (-1.64, 0.68) | 0.417 | 416 | -0.09 (-1.88, 1.70) | 0.923 |
| **DHT** | 959 | -0.11 (-1.07, 0.86) | 0.828 | 416 | -0.62 (-2.62, 1.38) | 0.542 |
| **Free DHT** | 941 | 0.04 (-0.90, 0.97) | 0.938 | 408 | -0.49 (-2.71, 1.73) | 0.666 |
| **Progesterone** | 959 | -0.27 (-1.30, 0.76) | 0.608 | 416 | 8.87 (-6.67, 24.41) | 0.264 |
| **17-OHP** | 959 | 0.26 (-0.71, 1.23) | 0.604 | 416 | -0.24 (-1.57, 1.10) | 0.728 |
| **SHBG** | 1003 | 0.60 (-0.48, 1.68) | 0.278 | 468 | -0.65 (-2.24, 0.94) | 0.425 |
| The full model was adjusted for age, smoking, physical activity, alcohol consumption, SBP, HDL-C, LDL-C, diabetes, antihypertensive medication and lipid lowering medication | | | | | | |
| Sex hormones were sex-specifically z-standardized prior to the analysis. The coefficient estimates represent the change of the outcomes corresponding to 1-standard deviation increase of the sex hormone.  P<0.0056 (0.05/9) is considered significant with Bonferroni correction for multiple testing.  *Abbreviations*: SBP, systolic blood pressure; HDL-C, high-density lipoprotein cholesterol; LDL-C, low-density lipoprotein cholesterol; DHEA, dehydroepiandrosterone; DHEAS, dehydroepiandrosterone-sulfate; DHT, dihydrotestosterone; SHBG, sex hormone-binding globulin; 17-OHP, 17α-hydroxyprogesterone; β, β-estimate. | | | | | | |

| **Supplementary Table 5**. **Association of endogenous sex hormone with fatty liver index with additional adjustment for CRP, TSH, serum albumin and SHBG.** | | | | | | |
| --- | --- | --- | --- | --- | --- | --- |
|  | **Men** | | | **Postmenopausal women** | | |
| ***Baseline FLI*** | | | | | | |
|  | N | **β, 95% CI** | *P value* | **N** | **β, 95% CI** | *P value* |
| **Testosterone** | 1308 | **-2.72 (-4.12, -1.32)** | **<0.001** | 647 | 1.39 (-0.45, 3.23) | 0.139 |
| **Free testosterone** | 1308 | **-2.37 (-3.68, -1.07)** | **<0.001** | 647 | 1.11 (-0.28, 2.50) | 0.119 |
| **DHEA** | 1308 | 0.10 (-1.31, 1.50) | 0.895 | 647 | -1.27 (-3.96, 1.41) | 0.353 |
| **DHEAS** | 1308 | -0.01 (-1.47, 1.45) | 0.985 | 647 | -0.24 (-2.65, 2.17) | 0.846 |
| **DHT** | 1308 | -1.17 (-2.43, 0.10) | 0.071 | 647 | -1.10 (-3.45, 1.25) | 0.359 |
| **Free DHT** | 1308 | -1.03 (-2.24, 0.18) | 0.096 | 647 | 0.06 (-2.38, 2.49) | 0.965 |
| **Progesterone** | 1308 | **-1.76 (-2.99, -0.52)** | **0.005** | 647 | 17.41 (-2.77, 37.59) | 0.091 |
| **17-OHP** | 1308 | **-2.36 (-3.58, -1.15)** | **<0.001** | 647 | 1.62 (-0.54, 3.78) | 0.141 |
| ***Follow-up FLI**** | | | | | | |
| **Testosterone** | 929 | 0.62 (-0.61, 1.85) | 0.321 | 397 | -0.44 (-2.33, 1.46) | 0.653 |
| **Free testosterone** | 929 | 0.61 (-0.47, 1.68) | 0.272 | 397 | -0.32 (-1.82, 1.18) | 0.677 |
| **DHEA** | 929 | -0.68 (-1.78, 0.41) | 0.221 | 397 | -1.10 (-3.16, 0.97) | 0.299 |
| **DHEAS** | 929 | -0.58 (-1.76, 0.61) | 0.341 | 397 | -0.26 (-2.15, 1.63) | 0.788 |
| **DHT** | 929 | -0.14 (-1.17, 0.89) | 0.791 | 397 | -0.53 (-2.61, 1.56) | 0.621 |
| **Free DHT** | 929 | 0.26 (-0.69, 1.20) | 0.596 | 397 | -0.86 (-3.19, 1.47) | 0.471 |
| **Progesterone** | 929 | -0.30 (-1.35, 0.76) | 0.583 | 397 | 10.31 (-5.70, 26.33) | 0.208 |
| **17-OHP** | 929 | 0.24 (-0.75, 1.23) | 0.636 | 397 | -0.22 (-1.59, 1.14) | 0.749 |
| Models were adjusted for age, smoking, physical activity, alcohol consumption, SBP, HDL-C, LDL-C, diabetes, antihypertensive medication, lipid lowering medication, CRP, TSH, serum albumin and SHBG.  *For the outcome of FLI at the follow-up, models were also adjusted for baseline FLI. | | | | | | |
| Sex hormones were sex-specifically z-standardized prior to the analysis. The coefficient estimates represent the change of the outcomes corresponding to 1-standard deviation increase of the sex hormone.  P<0.0056 (0.05/9) is considered significant with Bonferroni correction for multiple testing.  *Abbreviations*: FLI, fatty liver index; SBP, systolic blood pressure; HDL-C, high-density lipoprotein cholesterol; LDL-C, low-density lipoprotein cholesterol; CRP, C-reactive protein; TSH, Thyroid-stimulating hormone; DHEA, dehydroepiandrosterone; DHEAS, dehydroepiandrosterone-sulfate; DHT, dihydrotestosterone; SHBG, sex hormone-binding globulin; 17-OHP, 17α-hydroxyprogesterone; β, β-estimate. | | | | | | |

| **Supplementary Table 6. Mendelian randomization estimates of the relationship between sex hormones/SHBG sex-specific clusters and liver fat measured with MRI- hepatic proton density fat fraction.** | | | | | | | | | |
| --- | --- | --- | --- | --- | --- | --- | --- | --- | --- |
|  |  | **IVW** |  | **Weighted median** |  | **Weighted mode** |  | **MR-Egger** |  |
| **Exposure** | **N instruments** | **β (95% CI)** | ***P value*** | **β (95% CI)** | ***P value*** | **β (95% CI)** | ***P value*** | **β (95% CI)** | ***P value*** |
| **Male testosterone cluster** | 51 | -0.02 (-0.09, 0.05) | 0.586 | 0.01 (-0.09, 0.12) | 0.804 | 0.02 (-0.08, 0.12) | 0.680 | 0.03 (-0.08, 0.13) | 0.620 |
| **Male estradiol cluster** | 8 | -0.28 (-1.16, 0.59) | 0.525 | -0.06 (-0.77, 0.65) | 0.864 | 0.06 (-0.67, 0.80) | 0.872 | 1.39 (-1.68, 4.46) | 0.410 |
| **Female testosterone cluster** | 116 | -0.03 (-0.09, 0.03) | 0.331 | 0.03 (-0.04, 0.10) | 0.361 | 0.01 (-0.06, 0.09) | 0.728 | 0.02 (-0.08, 0.12) | 0.700 |
| **Male SHBG cluster** | 148 | **-0.20 (-0.34, -0.06)** | **0.005** | -0.09 (-0.21, 0.04) | 0.176 | -0.05 (-0.16, 0.06) | 0.354 | -0.11 (-0.32, 0.09) | 0.274 |
| **Female SHBG cluster** | 159 | **-0.43 (-0.61, -0.25)** | **<0.001** | **-0.18 (-0.31, -0.05)** | **0.006** | -0.18 (-0.32, -0.04) | 0.014 | -0.17 (-0.44, 0.11) | 0.233 |
| Clusters of genetic instruments with primary effects on specific sex hormone(s) were identified by Ruth et al. (2020) (PMID: 32042192). These included: (1) a male T cluster with primary increasing effect on T and secondary increasing effect on E2 but independent of SHBG; (2) a male SHBG cluster with primary SHBG increasing effect and secondary increasing effect on total T and decreasing effect on bioT as well as increasing effect on E2; (3) a male E2 cluster with only increasing effect on E2; (4) a female T cluster with primary T increasing effect independent of SHBG; (5) a female SHBG cluster with primary increasing effect on SHBG and secondary opposing effect on T and bioT.  Mendelian randomization analysis was carried out with the inverse-variance weighted approach as the main analysis, and robust methods such as weighted median, weighted mode and MR-Egger were carried out as sensitivity analyses. P<0.0071 (0.05/7) is considered significant with Bonferroni correction for multiple testing. *Abbreviations:* SHBG, sex hormone-binding globulin; IVW, inverse-variance weighted; T, testosterone; bioT, bioavailable testosterone; E2, estradiol; SHBG, sex hormone-binding globulin. | | | | | | | | | |

| **Supplementary Table 7. Mendelian randomization estimates of the relationship between sex hormones/SHBG and liver fat measured with MRI-hepatic proton density fat fraction after excluding potential pleiotropic SNPs associated with metabolic risk factors.** | | | | | | | | | | |
| --- | --- | --- | --- | --- | --- | --- | --- | --- | --- | --- |
|  |  |  | **IVW** |  | **Weighted median** |  | **Weighted mode** |  | **MR-Egger** |  |
| **Exposure** | **Sex** | **N instruments** | **β (95% CI)** | ***P value*** | **β (95% CI)** | ***P value*** | **β (95% CI)** | ***P value*** | **β (95% CI)** | ***P value*** |
| **Total testosterone** | men | 90 | -0.03 (-0.09, 0.04) | 0.439 | -0.05 (-0.12, 0.02) | 0.171 | -0.03 (-0.09, 0.04) | 0.406 | 0.01 (-0.09, 0.11) | 0.810 |
|  | women | 104 | -0.02 (-0.07, 0.03) | 0.449 | 0.004 (-0.07, 0.08) | 0.918 | 0.01 (-0.07, 0.08) | 0.821 | -0.02 (-0.11, 0.06) | 0.614 |
| **Bioavailable testosterone** | men | 57 | 0.003 (-0.06, 0.06) | 0.927 | 0.02 (-0.08, 0.12) | 0.715 | 0.02 (-0.08, 0.13) | 0.662 | 0.04 (-0.07, 0.14) | 0.496 |
|  | women | 73 | 0.08 (0.00, 0.17) | 0.055 | 0.10 (-0.001, 0.21) | 0.053 | 0.13 (0.02, 0.24) | 0.024 | 0.13 (-0.03, 0.28) | 0.110 |
| **Estradiol** | men | 4 | 0.01 (-0.64, 0.66) | 0.967 | -0.05 (-0.77, 0.67) | 0.891 | 0.05 (-0.77, 0.86) | 0.920 | 0.01 (-2.40, 2.42) | 0.994 |
| **SHBG** | men | 127 | -0.10 (-0.22, 0.02) | 0.099 | -0.09 (-0.21, 0.03) | 0.138 | -0.05 (-0.16, 0.05) | 0.335 | -0.04 (-0.22, 0.13) | 0.607 |
|  | women | 110 | -0.13 (-0.39, 0.13) | 0.322 | -0.17 (-0.31, -0.04) | 0.013 | -0.15 (-0.30, -0.01) | 0.042 | 0.01 (-0.38, 0.40) | 0.974 |
| Potential pleiotropic SNPs are SNPs that are more closely related to metabolic risk factors, including fasting glucose, type 2 diabetes, coronary artery disease, HDL-C, LDL-C, triglycerides, total-cholesterol, SBP, DBP, BMI and waist-to-hip ratio adjusted for BMI, than sex hormones identified by Steiger-filtering by Ruth et al. (2020) (PMID: 32042192).  Mendelian randomization analysis was carried out with the inverse-variance weighted approach as the main analysis, and robust methods such as weighted median, weighted mode and MR-Egger were carried out as sensitivity analyses.  P≤0.007 (0.05/7) is considered significant with Bonferroni correction for multiple testing.  *Abbreviations:* SBP, systolic blood pressure; DBP, diastolic blood pressure; BMI, body mass index; HDL-C, high-density lipoprotein cholesterol; LDL-C, low-density lipoprotein cholesterol; SHBG, sex hormone-binding globulin; IVW, inverse-variance weighted: SNP, single nucleotide polymorphism | | | | | | | | | | |


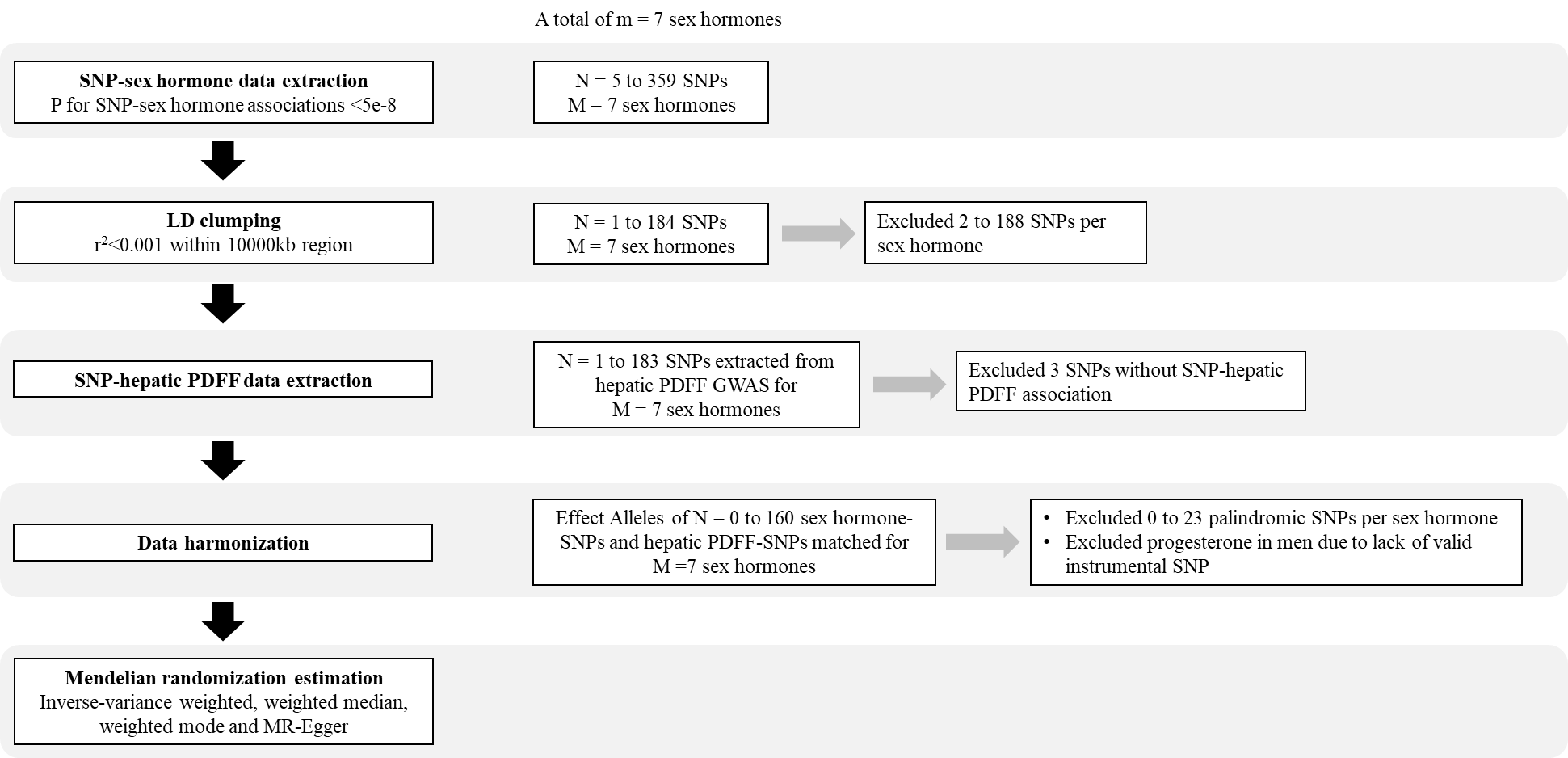


**Supplementary figure 1.** Schematic overview of the Mendelian randomization analysis

*Abbreviations*: SNP, single nucleotide polymorphism; LD, linkage disequilibrium; PDFF, proton density fat fraction; GWAS, genome-wide association analysis; MR, Mendelian randomization.

| **Men** | **Postmenopausal women** |
| --- | --- |
| 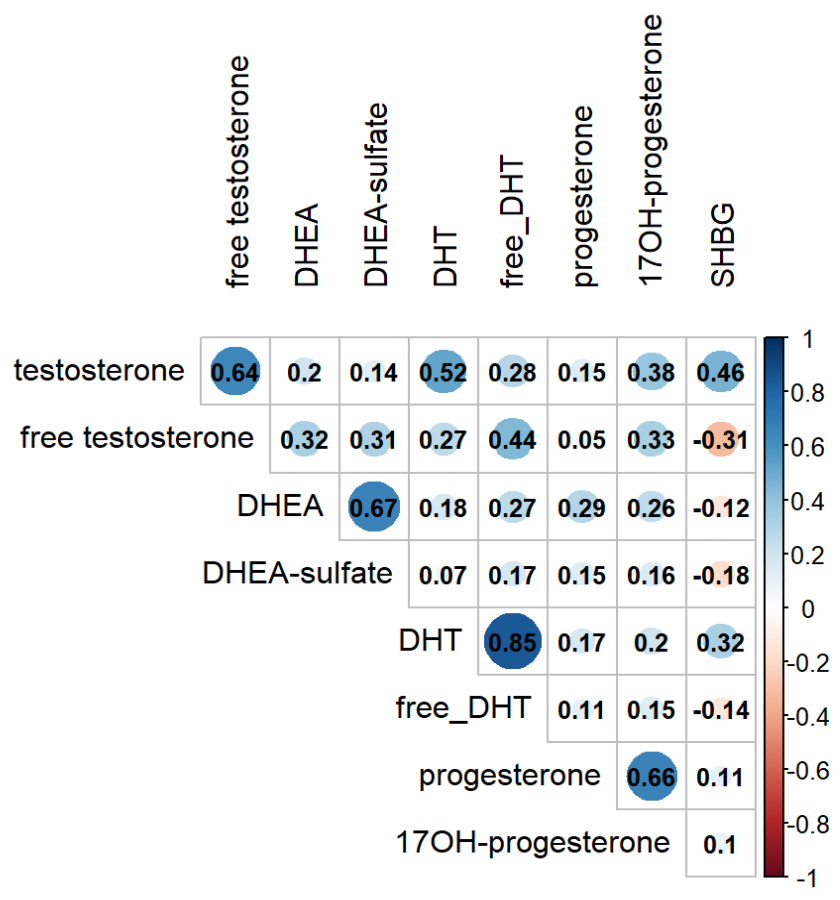 | 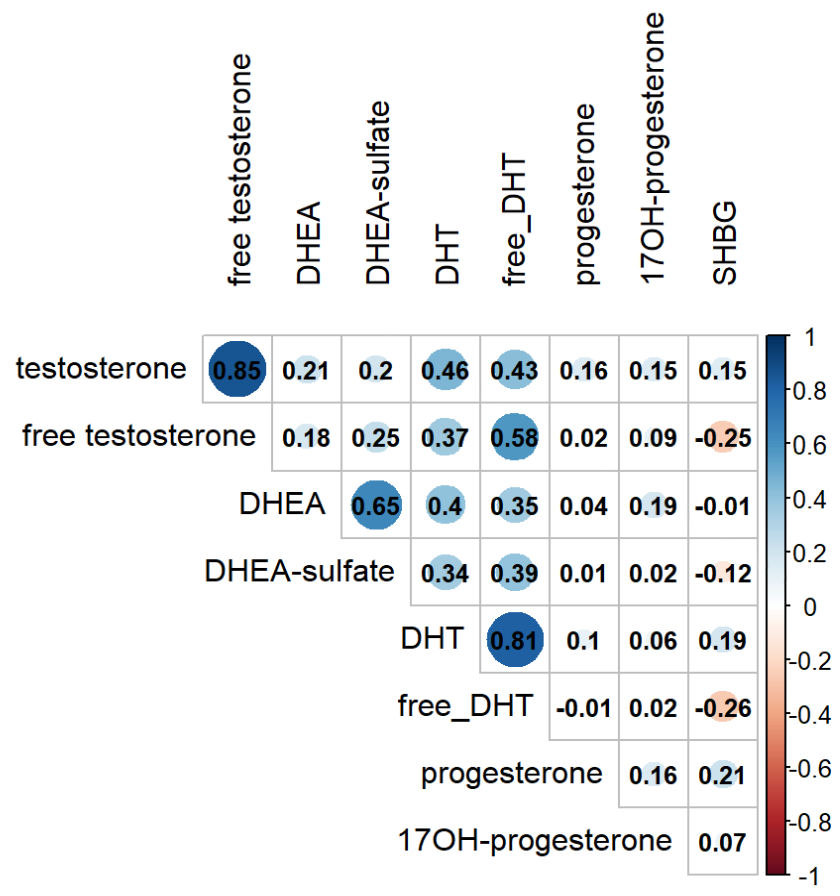 |

**Supplementary figure 2.** Sex-specific correlation matrix of the sex hormones

*Abbreviations:* DHEA, dehydroepiandrosterone; DHT, dihydrotestosterone; 17OH-progesterone, 17α-hydroxyprogesterone; SHBG, sex hormone-binding globulin.
